# Synthesis of fully bio-based poly (3-hydroxybutyrate)-oligo-2-ethyl oxazoline conjugates

**DOI:** 10.3389/fchem.2024.1367451

**Published:** 2024-03-14

**Authors:** Baki Hazer, Özlem Altunordu Kalaycı, Fatma Koçak

**Affiliations:** ^1^ Department of Aircraft Airframe Engine Maintenance, Kapadokya University, Ürgüp, Türkiye; ^2^ Departments of Chemistry/Nano Technology Engineering, Zonguldak Bülent Ecevit University, Zonguldak, Türkiye; ^3^ Departments of Physics, Zonguldak Bülent Ecevit University, Zonguldak, Türkiye

**Keywords:** bacterial polyester, PHB, oligo oxazoline, 2-ethyl oxazoline, cationic polymerization, two carboxylic acid-terminated PHB

## Abstract

This work refers to the synthesis and characterization of poly (3-hydroxybutyrate)-b-oligo (2-ethyl oxazoline) (oligoEtOx). Cationic ring-opening polymerization of 2-ethyl oxazoline yielded poly (2-ethyl oxazoline) (oligoEtOx) with a hydroxyl end. Carboxylic acid-terminated PHB was reacted with oligoEtOx via dicyclohexylcarbodiimide chemistry to obtain PHB-b-oligoEtOx conjugates. The obtained PHB-b-oligoEtOx conjugates were successfully characterized by ^1^H- and ^13^C NMR, FTIR, DSC, and size exclusion chromatography. PHB-b-oligoEtOx conjugates can be promising biologic active materials.

## Introduction

Poly (3-hydroxybutyrate) (PHB) is a microbial aliphatic biopolyester which is accumulated in bacterium cells from some carbon substrates (Ashby and Foglia, 1998; Kocer et al., 2003; Hazer and Steinbüchel, 2007; Chen, 2009; Ashby et al., 2019; Choi et al., 2020; Guzik et al., 2020; Bedade et al., 2021; Kacanski et al., 2023).

PHB is a crystalline polymer with melting transition (Tm) at approximately 170°C. It can also be synthesized by the anionic ring-opening polymerization of beta-butyrolactone (Hazer, 1996; Arkin et al., 2001).

The synthetic PHB is in R, S configuration, while bacterial PHB is only in R configuration (Caputo et al., 2022).

PHB modification reactions are important to prepare new PHB derivatives for some industrial and medical applications (Hazer, 2010; Hazer et al., 2012; Guennec et al., 2021). Some of them are halide derivatives (Arkin and Hazer, 2002; Yalcin et al., 2006; Erol et al., 2020), chitosan derivatives (Arslan et al., 2007), diethanol amine derivatives (Tuzen et al., 2016), trithiocarbonate derivatives (Hazer et al., 2020), methyl salicylate derivatives (Hazer et al., 2021), ricinoleic acid derivatives (Ullah et al., 2024), PEG derivatives (Hazer et al., 1999; Wadhwa et al., 2014), and caffeic acid derivatives (Abdelmalek et al., 2023).

Poly (2-ethyl-2-oxazoline) (oligoEtOx) is obtained by the cationic polymerization of 2-ethyl oxazoline (2-EtOx). OligoEtOx is a water-soluble polymer and is very popular in the field of biomedical and pharmaceutical applications (Vergaelen et al., 2023). Dual initiator techniques, including the carbocationic method and free radical polymerization, can be used to synthesize block copolymers (Hazer, 1991; Christova et al., 1997). In this manner, poly (2-ethyl-2-oxazoline) derivatives were successfully synthesized by polymer chemists for medical applications (Miyamoto et al., 1989; Christova et al., 2002; Diab et al., 2004; Park et al., 2004; Hoogenboom et al., 2005; Becer et al., 2008; Li et al., 2021; Göppert et al., 2023).

Very recently, Becer et al. reported the synthesis of poly (2-ethyl oxazoline)-b-poly (acrylate) hybrid multiblock copolymers via a click reaction. They evaluate their self-assembly behavior into stomatocyte-like nanoparticles (Hayes et al., 2023). The multiamide structure of polyEtOx makes it a candidate to mimic peptides, and it shows an antibacterial effect against *Staphylococcus aureus* (Hoogenboom, 2009).

Poly (2-ethyl oxazoline) is a new class of functional peptide that mimics with potential in a variety of biological applications (Zhou et al., 2020). PolyEtOx is a thermosensitive polymer with a lower critical solution temperature (LCST), changing the aqueous solution temperature at approximately 62°C (Christova et al., 2003; Park and Kataoka, 2007; Obeid et al., 2009; Hoogenboom and Schlaad, 2011).

Winnik et al. reported the cloud point of aqueous methyl poly(I-propyl oxazoline) with Mn 10 K g/mol. Turbidity decreases with the increasing concentration from ∼48°C to ∼39°C.

Block copolymers containing hydrophilic and hydrophobic blocks gain the properties of both related blocks. These different polymer blocks can be arranged linearly or as brush-type copolymers (Minoda et al., 1990; Xu et al., 1991; Förster and Antonietti, 1998; Bronstein et al., 1999; Chen et al., 1999; Hu et al., 2008; Mai and Eisenberg, 2012; Kalayci et al., 2013; Glaive et al., 2024; Hosseini et al., 2024; Wang et al., 2024).

The insertion of the hydrophilic polymer in a block copolymer will improve the colloidal stability of the nanoparticles for biomedical applications (Balcı et al., 2010; Kalaycı et al., 2010; Karahaliloğlu et al., 2020; Wen et al., 2023; Kilicay et al., 2024).

PHB is a commercially available biodegradable natural aliphatic polyester for some biomedical applications, such as implant biomaterials, tissue engineering, and food packaging applications (Chen and Zhang, 2018; Mehrpouya et al., 2021; Abdelmalek et al., 2023). PHB derivatives can be used as novel biodegradable adsorbents for analytical applications (Wadhwa et al., 2014; Unsal et al., 2015; Tuzen et al., 2016; Altunay et al., 2020; Ullah et al., 2024; Ali et al., 2024) for drug delivery systems (Bayram et al., 2008; Kılıcay et al., 2011; Kilicay et al., 2024).

In this work, we report the synthesis of poly (3-hydroxybutyrate)-oligo-2-ethyl oxazoline, fully bio-based amphiphilic polymer conjugates. Two carboxyl-terminated PHB were synthesized by refluxing PHB with adipic acid in the presence of Stannous octoate. Then, the carboxyl-terminated PHB was reacted with the hydroxyl end of oligooxazoline, which was obtained by the ring-opening cationic polymerization of 2-ethyl oxazoline. The physicochemical characterization of the PHB-oligo-2-ethyl oxazoline conjugates was carried out in detail.

## Experiment

### Materials

2-Ethyl oxazoline (2-EtOx) was supplied from Sigma-Aldrich and was passed into the Al_2_O_3_ column before use. N, N′-Dicyclohexylcarbodiimide (DCC; 99%), dimethylaminopyridine (DMAP; 99%), stannous 2-ethylhexanoate (Sn-oct; ≥92.5%), methyl p-toluene sulfonate (MepTs), and all other chemicals were purchased from Sigma-Aldrich. Poly (3-hydroxybutyrate) (PHB) and microbial polyester (Mn 187,000 g/mol, Mw/Mn 2.5, Biomer Inc.) were supplied from Biomer (Germany) (Neugebauer et al., 2007).

### Synthesis of oligo(2-ethyl oxazoline) (oligoEtOx)

2-Ethyl oxazoline was oligomerized by ring-opening cationic polymerization. A mixture of 2-ethyl oxazoline (2.01 g) and MepTs (0.20 g) as the catalyst was dissolved in acetonitrile (AcCN, 2.0 mL) in a reaction bottle. Argon was passed through the solution for 2 min. Polymerization was carried out at 100°C for 70 min. The polymer precipitated in excess diethyl ether. It was dried under vacuum at 40°C for 24 h (yield: 1.98 g, Mn 900 g/mol, and PDI: 1.57).

### Synthesis of dicarboxylic acid-terminated PHB, PHB-COOH

A mixture of adipic acid (1.00 g), PHB (0.64 g), and Sn-oct (20 mg) was dissolved in CHCl_3_ (20 mL). It was refluxed at 85°C for 4 h. After half of the solvent was evaporated, the product was precipitated from excess methanol and dried under vacuum at 40°C for 24 h. The yield was 0.86 g.

### Characterization

^1^H NMR spectra were taken with an Agilent NMR 600 MHz NMR (Agilent, Santa Clara, CA, United States) spectrometer equipped with a 3-mm broadband probe. FT-IR spectra of the substituted polymer samples were recorded using a Bruker Model, Tensor II instrument with the ATR technique in the transmissive mode and a scan rate of 4,000 to 450 cm^−1^. A Viscotek GPCmax autosampler system, consisting of a pump, three ViscoGEL GPC columns (G2000H HR, G3000H HR, and G4000H HR), and a Viscotek differential refractive index (RI) detector, was used to determine the molecular weights of the polymer products. A calibration curve was generated with five polystyrene (PS) standards of molecular weight 2,960, 8,450, 50,400, 200,000, and 696,500 Da with low polydispersity. Data were analyzed using Viscotek OmniSEC Omni 01 software. Differential scanning calorimetry (DSC) was used in the thermal analysis of the obtained polymers. The DSC analysis was carried out under nitrogen using a TA Q2000 DSC instrument that was calibrated using indium (Tm = 156.6°C) and a Q600 Simultaneous DSC-TGA (SDT) series thermal analysis system. DSC measures the temperatures and heat flows associated with thermal transitions in the polymer samples obtained. The dried polymer samples were heated from −60°C to 220°C under a nitrogen atmosphere. All melting endotherms (Tm) were reported as peak temperatures, while all glass transition temperatures (Tg) were reported as midpoint temperatures. Thermogravimetric analysis (TGA) was used to determine the decomposition temperature (T_d_) characteristics of the polymers by measuring the weight loss under a nitrogen atmosphere over time. In these analyses, the obtained polymers were heated from 20°C to 600°C at a rate of 10°C/min, and the results were determined based on the first derivative of each curve. Scanning electron microscopy (SEM) imaging (Zeiss EVO lS10) was used for the characterization of the obtained polymers.

## Results and discussion

Ring-opening cationic polymerization of 2-ethyl oxazoline, in the presence of MepTs, yielded oligo(2-ethyl oxazoline) (OligoOx). Oxazoline oligomers were obtained in several types, with molar masses changing from 700 to 900 g/mol. Characterization of OligoEtOx confirmed the polymer structure. The FTIR spectrum contained the characteristic signals at 3,462 cm^−1^, 2,977–2,939 cm^−1^, 1,624 cm^−1^, and 1,187 cm^−1^ related to –OH, -C-H, amid carbonyl, and –N-CH_2_- groups, respectively. Typical characteristic groups were also observed in the ^1^H NMR spectrum at chemical shifts at 3.5 ppm (-N-C**H**_2_-), 2.2–2.5 ppm (-C**H**_2_-C(O)-), and 1.1 ppm (C**H**_3_-CH_2_-).

**FIGURE 1:**
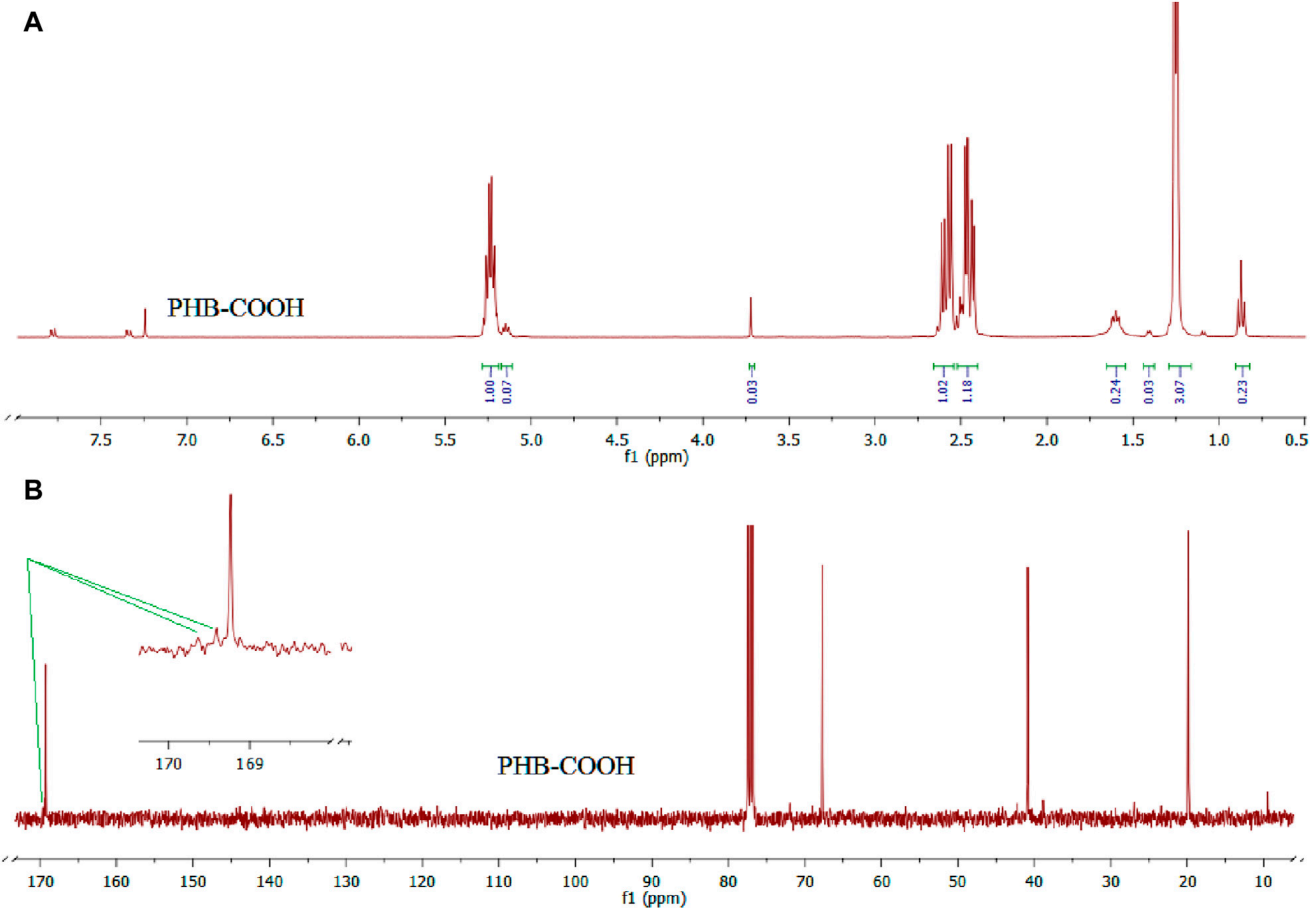
1H **(A)** and 13C **(B)** NMR spectra of the PHB-COOH sample.

PHB with two carboxylic acid terminals was obtained by the reaction of an equimolar amount of adipic acid and PHB under reflux conditions at 85°C. The characteristic signals were observed in the 1H NMR and 13C NMR spectra of the as-synthesized PHB-COOH sample, which is seen in Figure 1, including 1H (a) and 13C (b) NMR spectra. 1H NMR, δ _(ppm)_: 1.3 ppm for –CH_3_, 1.5 ppm for –CH_2_-CH_2_-, 2.4–2.6 ppm for –CH_2_-COO-, 3.7 ppm for CH_2_-OC(O)CH_2_–, and 5.1–5.3 ppm for–CHO–. 13C NMR, δ_(ppm)_: 10 (–CH_2_-CH_2_-), 20 (CH_3_-), 40 (-CH_2_-C(O)-, 67 (-CH-O-), 169.1, and 169.2 carbonyls for PHB and adipic acid moieties, respectively.

### Synthesis of PHB-oligoEtOx polymer conjugates

**FIGURE 2:**
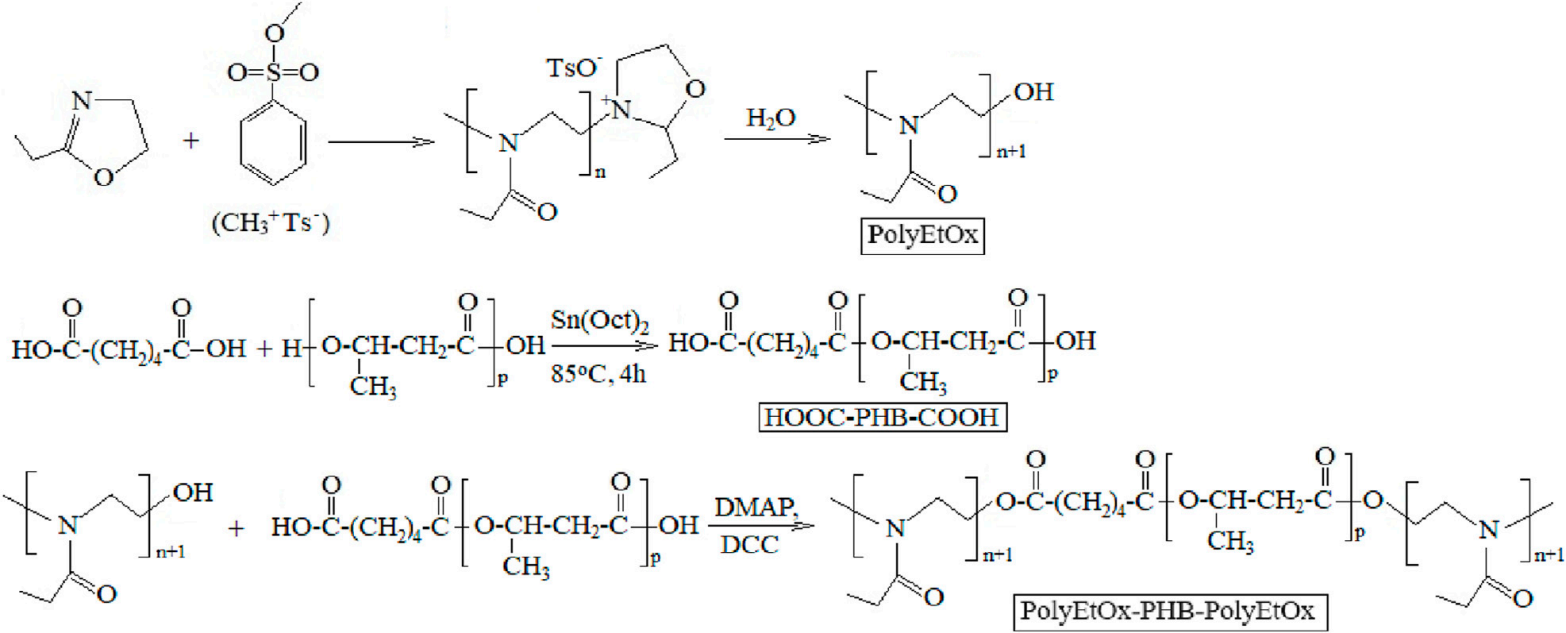
Reaction pathways of the synthesis of the oligoEtOx-b-PHB block copolymer.

OligoEtOx was capped with the carboxylic acid ends of PHB-COOH to produce the novel PHB-b-oligoEtOx block copolymer. The reaction pathways can be seen in Figure 2.

The reaction conditions and results are listed in Table 1. Changing the feed percentage of oligoEtOx from 14% to 41% against PHB(COOH)_2_ was reacted at room temperature. The yield of the obtained block copolymer was gravimetrically determined. The polymer obtained was precipitated from the acidified diethyl ether and dried in vacuum. For further purification, it was soaked in distilled water for 24 h in order to remove the unreacted oligoEtOx residue.

**TABLE 1:** Synthesis conditions and results of the PHB-b-oligoEtOx block copolymer at room temperature for 24 h.

Code	PHB(COOH)_2_ (g)	PolyOx (g) (%)	DMAP (g)	DCC (g)	Yield (g) (%)
PHB-Ox-21	1.08	0.18 14	0.044	0.82	1.04 83
PHB-Ox-23	2.02	0.67 25	0.242	5.03	2.24 83
PHB-Ox-22	1.08	0.56 34	0.092	2.45	1.19 73
PHB-Ox-24	2.02	1.41 41	0.131	3.12	2.34 68

**FIGURE 3:**
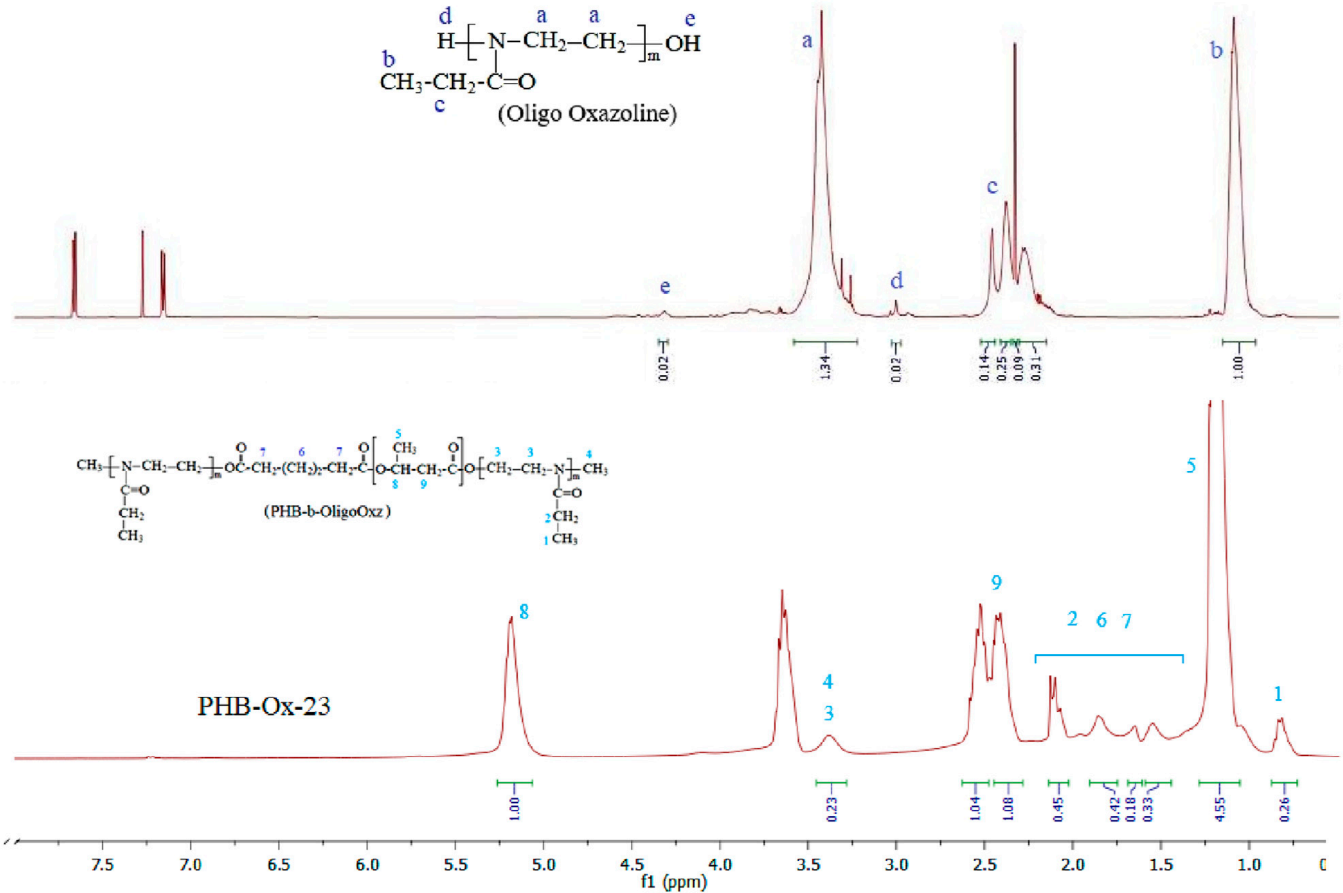
^1^H NMR spectra of the oligooxazoline and the as-synthesized PHB-oligoEtOx-23 conjugate in CDCl_3_.

Characterization of PHB-oligoEtOx conjugates was carried out by ^1^H and ^13^C NMR, FTIR, differential scanning calorimeter (DSC), and thermo-gravimetric analysis (TGA) techniques. PHB-Ox-21, −22, −23, and −24 samples contained the characteristic samples of oligoEtOx blocks at 3.4 and 3.6 ppm related to the –C**H**_2_-N- groups. Chemical shifts at 1.2 ppm (C**H**_2_-CH_2_-) and 2.5 ppm (-C**H**_2_-C(O)-) were overlapped with those of PHB blocks. The amount of oligoOx blocks in the obtained PHB-oligoEtOx −21, −22, −23, and −24 conjugates was calculated while comparing them with integral values of the signals at 5.2 ppm (PHB) and 3.5 ppm (oligoOx) 19, 52, 17, and 14%, respectively. Figure 3 shows the ^1^H NMR spectra of the comparison of oligoOx with the as-synthesized PHB-oligoOx-23 conjugate in CDCl_3_.

**FIGURE 4:**
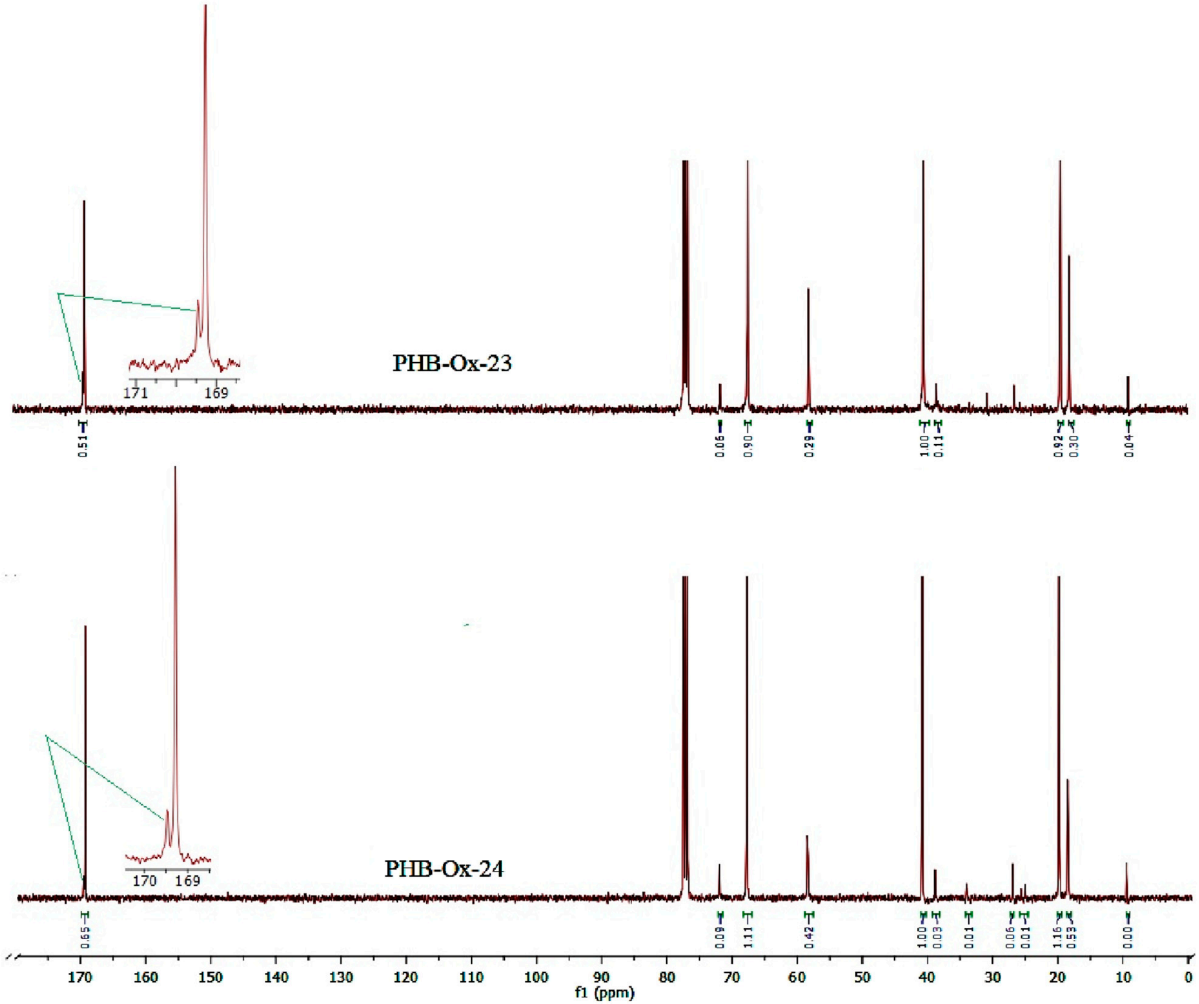
13C NMR spectra of the as-synthesized PHB-oligEtOx-23, -24 block copolymers in CDCl_3_.

^13^C NMR spectra of the PHB-oligoEtOx-23, -24 block copolymers contained the characteristic signals of the PHB and oligoOx blocks. Chemical shifts: 19, 20 ppm (-**C**H_3_, PHB, oligoEtOx), 39, 40 ppm (-**C**H_2_-C(O)-, PHB, oligoEtOx), 58 ppm (-N-**C**H_2_-, oligoEtOx), 67 ppm (-**C**H-O-, PHB), and 169.1 and 169.2 ppm (-**C**=O, PHB and oligoOx). Figure 4 shows the ^13^C NMR spectra of the as-synthesized PHB-oligoEtOx-23 and −24 conjugates in CDCl_3_.

**FIGURE 5:**
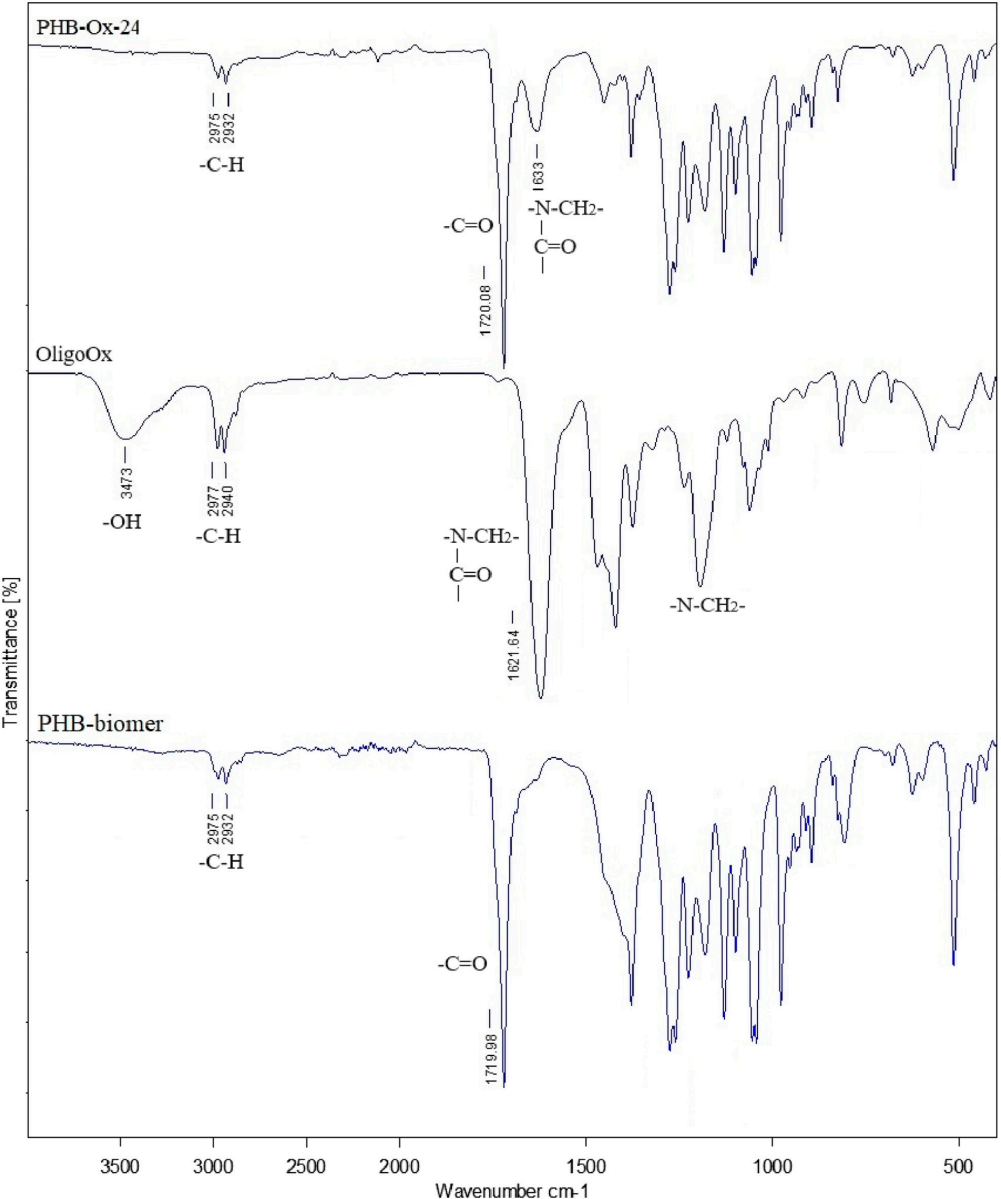
FTIR spectrum of PHB-oligEtOx-24 compared with the pristine oligEtOx.

Typical FTIR spectra of PHB-oligoOx-24, oligoOx, and pristine PHB compared with each other are shown in Figure 5. The typical characteristic signal of the oligoEtOx block was observed at 1,633 cm^−1^ related to the –N-C(O) group. The signals of the characteristic groups were marked on the related spectra.

**FIGURE 6:**
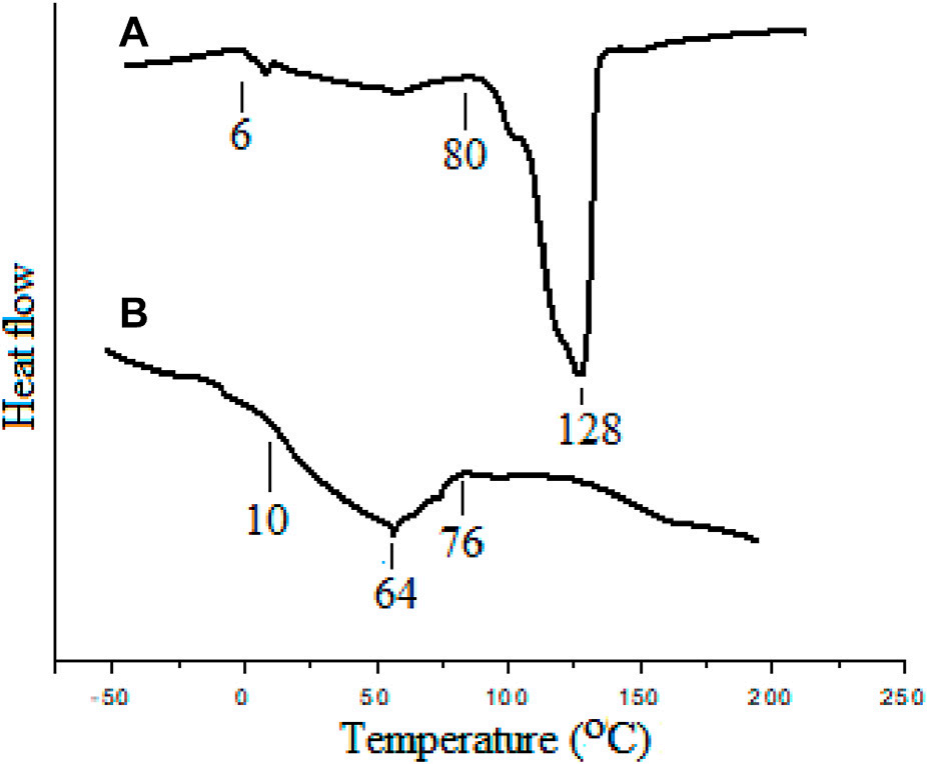
DSC curves of **(A)** PHB-oligoOx-21 and **(B)** oligoOx.

Thermal properties of the block copolymers were measured using a differential scanning calorimeter (DSC). The oligoEtOx sample has a wide glass transition (Tm) between 10 and 76°C and the maximum at 64°C. In the PHB-oligoEtOx polymer conjugate, the same wide melting transition between 6 and 80°C together with that of PHB at 128°C was observed. The PHB homopolymer has a melting transition at 170°C. The lower melting transition of the PHB block in the copolymer shows the plasticizing effect of oligoEtOx. Figure 6 shows the DSC curves of PHB-oligEtOx-21 and homo oligoOx. Homo oligoEtOx showed the glass transition temperature (Tg) at 10°C.

**FIGURE 7:**
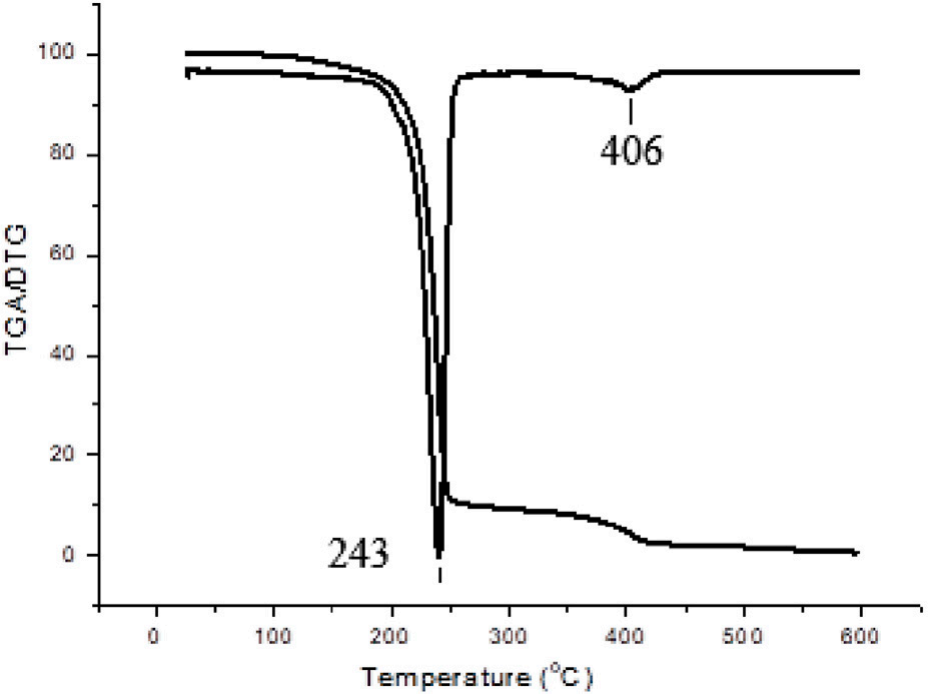
TGA/DTA curves of the PHB-oligoEtOx-22 conjugate.

TGA analysis was done in the PHB-oligoEtOx conjugates. The all TGA/DTG curves contained two decomposition temperatures (Td)s: 243 and 406°C (for PHB-oligoOx-22), 249 and 381°C (for PHB-oligoOx-23), and 247 and 381°C (for PHB-oligoEtOx-24). The TGA/DTA curves of the PHB-oligoEtOx-22 conjugate are given in Figure 7. Decomposition of the PHB blocks changes between 243 and 249°C, while that of the oligo oxazoline blocks changes between 386 and 406°C (Bouten et al., 2015).

## Conclusion

A fully biodegradable amphiphilic copolymer was obtained in this work. The hydroxyl end of oligoEtOx can easily be reacted with some other reagents to obtain polyoxazoline derivatives. Water-soluble hydrophilic oligoEtOx makes the hydrophobic polymers amphiphilic, which can be useful for medical applications. Combining natural and biodegradable hydrophobic properties of PHB with hydrophilic oligoEtOx yields a novel amphiphilic natural biopolymer.

Block copolymers containing hydrophilic and hydrophobic blocks gain the unique properties of both the related blocks. These different polymer blocks can be arranged linearly or as brush-type copolymers. The insertion of the hydrophilic polymer in a block copolymer can improve the colloidal stability of the biologic active nanoparticles for biomedical applications. Therefore, the PHB-b-oligoEtOx block copolymer can be a promising biopolymer for medical applications.

## Data Availability

The original contributions presented in the study are included in the article/Supplementary material; further inquiries can be directed to the corresponding author.

## References

[B1] Abdelmalek F. Rofeal M. Pietrasik J. Steinbüchel A. (2023). Novel biodegradable nanoparticulate chain-end functionalized polyhydroxybutyrate–caffeic acid with multifunctionalities for active food coatings. ACS Sustain. Chem. Eng. 11 (18), 7123–7135. 10.1021/acssuschemeng.3c00389 37180027 PMC10171369

[B2] Ali J. Tuzen M. Jatoi W. Hazer B. Feng X. (2024). A novel block copolymer containing gadolinium oxide nanoparticles in ultrasound assisted-dispersive solid phase microextraction of total arsenic in human foodstuffs: a multivariate optimization methodology. Food Chem. 437, 137908. 10.1016/j.foodchem.2023.137908 37925781

[B3] Altunay N. Tuzen M. Hazer B. Elik A. (2020). Usage of the newly synthesized Poly(3-hydroxy butyrate)-b-poly(vinyl benzyl xanthate) block copolymer for vortex-assisted solid-phase microextraction of cobalt (II) and nickel (II) in canned foodstuffs. Food Chem. 321, 126690. 10.1016/j.foodchem.2020.126690 32244139

[B4] Arkin A. H. Hazer B. (2002). Chemical modification of chlorinated microbial polyesters. Biomacromolecules 3 (6), 1327–1335. 10.1021/bm020079v 12425672

[B5] Arkin A. H. Hazer B. Adamus G. Kowalczuk M. Jedlinski Z. Lenz R. W. (2001). Synthesis of poly(2-methyl-3-hydroxyoctanoate) via anionic polymerization of α-Methyl-β-pentyl-β-propiolactone. Biomacromolecules 2 (3), 623–627. 10.1021/bm015528q 11710013

[B6] Arslan H. Hazer B. Yoon S. C. (2007). Grafting of poly(3-hydroxyalkanoate) and linoleic acid onto chitosan. J. Appl. Polym. Sci. 103, 81–89. 10.1002/app.24276

[B7] Ashby R. D. Foglia T. A. (1998). Poly(hydroxyalkanoate) biosynthesis from triglyceride substrates. Appl. Microbiol. Biotechnol. 49, 431–437. 10.1007/s002530051194

[B8] Ashby R. D. Solaiman D. K. Strahan G. D. (2019). The Use of Azohydromonas lata DSM 1122 to produce 4-hydroxyvalerate-containing polyhydroxyalkanoate terpolymers, and unique polymer blends from mixed-cultures with Burkholderia sacchari DSM 17165. J. Polym. Environ. 27, 198–209. 10.1007/s10924-018-1332-2

[B9] Balcı M. Allı A. Hazer B. Guven O. Cavicchi K. Cakmak M. (2010). Synthesis and characterization of novel comb-type amphiphilic graft copolymers containing polypropylene and polyethylene glycol. Polym. Bull. 64, 691–705. 10.1007/s00289-009-0211-3

[B10] Bayram C. Denkbas E. B. Kiliçay E. Hazer B. Çakmak H. B. Noda I. (2008). Preparation and characterization of triamcinolone acetonide-loaded poly(3-hydroxybutyrate-co-3-hydroxyhexanoate) (PHBHx) microspheres. J. Bioact. Compat. Polym. 23, 334–347. 10.1177/0883911508092790

[B11] Becer C. R. Paulus R. M. Hoppener S. Hoogenboom R. Fustin C.-A. Gohy J.-F. (2008). Synthesis of poly(2-ethyl-2-oxazoline)-b-poly(styrene) copolymers via a dual initiator route combining cationic ring-opening polymerization and atom transfer radical polymerization. Macromolecules 41, 5210–5215. 10.1021/ma800527h

[B12] Bedade D. K. Edson C. B. Gross R. A. (2021). Emergent approaches to efficient and sustainable polyhydroxyalkanoate production. Molecules 26 (11), 3463. 10.3390/molecules26113463 34200447 PMC8201374

[B13] Bouten P. J. M. Lava K. van Hest J. C. M. Hoogenboom R. (2015). Thermal properties of methyl ester-containing poly(2-oxazoline)s. Polymers 7, 1998–2008. 10.3390/polym7101494

[B14] Bronstein L. Kramer E. Berton B. Burger C. Forster S. Antonietti M. (1999). Successive use of amphiphilic block copolymers as nanoreactors and templates: preparation of porous silica with metal nanoparticles. Chem. Mater 11, 1402–1405. 10.1021/cm980762h

[B15] Caputo M. R. Tang X. Westlie A. H. Sardon H. Chen E. Y.-X. Müller A. J. (2022). Effect of chain stereoconfiguration on poly(3-hydroxybutyrate) crystallization kinetics. Biomacromolecules 23, 3847–3859. 10.1021/acs.biomac.2c00682 35929661 PMC9472230

[B16] Chen G. Q. (2009). A microbial polyhydroxyalkanoates (PHA) based bio- and materials industry. Chem. Soc. Rev. 38 (8), 2434–2446. 10.1039/b812677c 19623359

[B17] Chen G.-Q. Zhang J. (2018). Microbial polyhydroxyalkanoates as medical implant biomaterials. Artif. Cells Nanomed. Biotechnol. 46 (1), 1–18. 10.1080/21691401.2017.1371185 28849679

[B18] Chen M.-Q. Serizawa T. Akashi M. (1999). Graft copolymers having hydrophobic backbone and hydrophilic branches. xvi. Polystyrene microspheres with poly(N-isopropylacrylamide) branches on their surfaces: size control factors and thermosensitive behavior. Polym. Adv. Technol. 10, 120–126. 10.1002/(sici)1099-1581(199901/02)10:1/2<120::aid-pat781>3.3.co;2-7

[B19] Choi S. Y. Cho I. J. Lee Y. Kim Y. J. Kim K. J. Lee S. Y. (2020). Microbial polyhydroxyalkanoates and nonnatural polyesters. Adv. Mat. 32, 1907138. 10.1002/adma.201907138 32249983

[B20] Christova D. Velichkova R. Goethals E. J. (1997). Bis-macromonomers of 2-alkyl-2-oxazolines - synthesis and polymerization. Macromol. Rapid Commun. 18, 1067–1073. 10.1002/marc.1997.030181210

[B21] Christova D. Velichkova R. Goethals E. J. Du Prez F. E. (2002). Amphiphilic segmented polymer networks based on poly(2-alkyl-2-oxazoline) and poly(methyl methacrylate). Polymer 43, 4585–4590. 10.1016/s0032-3861(02)00313-0

[B22] Christova D. Velichkova R. Loos W. Goethals E. J. Du Prez F. (2003). New thermo-responsive polymer materials based on poly(2-ethyl-2-oxazoline) segments. Polymer 44, 2255–2261. 10.1016/s0032-3861(03)00139-3

[B23] Diab C. Akiyama Y. Kataoka K. Winnik F. M. (2004). Microcalorimetric study of the temperature-induced phase separation in aqueous solutions of poly(2-isopropyl-2-oxazolines). Macromolecules 37, 2556–2562. 10.1021/ma0358733

[B24] Erol A. Rosberg D. B. H. Hazer B. Göncü B. S. (2020). Biodegradable and biocompatible radiopaque iodinated poly-3-hydroxy butyrate. synthesis, characterization and *in vitro/in vivo* x-ray visibility. Polym. Bull. 77, 275–289. 10.1007/s00289-019-02747-6

[B25] Förster S. Antonietti M. (1998). Amphiphilic block copolymers in structure-controlled nanomaterial hybrids. Adv. Mater. 10, 195–217. 10.1002/(sici)1521-4095(199802)10:3<195::aid-adma195>3.0.co;2-v

[B26] Glaive A.-S. Le Cœur C. Guigner J.-M. Amiel C. Volet G. (2024). Amphiphilic heterograft copolymers bearing biocompatible/biodegradable grafts. Gisèle Volet Langmuir 40 (4), 2050–2063. 10.1021/acs.langmuir.3c02772 38243903

[B27] Göppert N. E. Quader S. Van Guyse J. F. R. Weber C. Kataoka K. Schubert U. S. (2023). Amphiphilic poly(2-oxazoline)s with glycine-containing hydrophobic blocks tailored for panobinostat- and imatinib-loaded micelles. Biomacromolecules 24, 5915–5925. 10.1021/acs.biomac.3c00934 37987713

[B28] Guennec A. Brelle L. Balnois E. Linossier I. Renard E. Langlois V. (2021). Antifouling properties of amphiphilic poly(3-hydroxyalkanoate): an environmentally-friendly coating. Biofouling 37 (8), 894–910. 10.1080/08927014.2021.1981298 34579623

[B29] Guzik M. Witko T. Steinbuchel A. Wojnarowska M. Soltysik M. Wawak S. (2020). What has been trending in the research of polyhydroxyalkanoates? A systematic review. Front. Bioengin Biotechnol. 8, 959. 10.3389/fbioe.2020.00959 PMC7513618 33014998

[B30] Hayes G. Drain B. Lefley J. Becer C. R. (2023). Hybrid multiblock copolymers of 2-oxazoline and acrylates via Cu-catalyzed Azide−Alkyne cycloaddition step-growth mechanism. Macromolecules 56 (1), 271–280. 10.1021/acs.macromol.2c01865

[B31] Hazer B. (1991). Synthesis of tetrahydrofuran-styrene (or methyl methacrylate) block copolymers via cationic-to-radical transformation. Eur. Polym. J. 27, 775–777. 10.1016/0014-3057(91)90007-b

[B32] Hazer B. (1996). Poly(β‐hydroxynonanoate) and polystyrene or poly(methyl methacrylate) graft copolymers: microstructure characteristics and mechanical and thermal behavior. Macromol. Chem. Phys. 197, 431–441. 10.1002/macp.1996.021970202

[B33] Hazer B. (2010). Amphiphilic poly (3-hydroxy Alkanoate)s: potential candidates for medical applications. Int. J. Polym. Sci. 2010, 1–8. 10.1155/2010/423460

[B34] Hazer B. Eren M. Senemoğlu Y. Modjinou T. Renard E. Langlois V. (2020). Novel poly(3-hydroxy butyrate) macro raft agent. Synthesis and characterization of thermoresponsive block copolymers. J. Polym. Res. 27, 147. 10.1007/s10965-020-02133-1

[B35] Hazer B. Lenz R. W. Çakmaklı B. Borcaklı M. Koçer H. (1999). Preparation of poly(ethylene glycol) grafted poly(3-hydroxyalkanoate) networks. Macromol. Chem. Phys. 200, 1903–1907. 10.1002/(SICI)1521-3935(19990801)200:8<1903::AID-MACP1903>3.0.CO;2-J

[B36] Hazer B. Steinbüchel A. (2007). Increased diversification of polyhydroxyalkanoates by modification reactions for industrial and medical applications. Appl. Microbiol. Biotechnol. 74 (1), 1–12. 10.1007/s00253-006-0732-8 17146652

[B37] Hazer B. Subramaniyan S. Zhang B. (2021). Synthesis of biobased block copolymers using A novel methacrylated methyl salicylate and poly(3‐hydroxybutyrate). ChemistrySelect 6, 12255–12265. 10.1002/slct.202102977

[B38] Hazer D. B. Kilicay E. Hazer B. (2012). Poly(3-hydroxyalkanoate)s: diversification and biomedical applications. Mater Sci. Eng. C 32 (4), 637–647. 10.1016/j.msec.2012.01.021

[B39] Hoogenboom R. (2009). Poly(2-oxazoline)s: a polymer class with numerous potential applications. Angew. Chem. Int. Ed. 48, 7978–7994. 10.1002/anie.200901607 19768817

[B40] Hoogenboom R. Paulus R. M. Fıjten M. W. M. Schubert U. S. (2005). Concentration effects in the cationic ring-opening polymerization of 2-ethyl-2-oxazoline in N,N dimethylacetamide. J. Polym. Sci. Part A Polym. Chem. 43, 1487–1497. 10.1002/pola.20603

[B41] Hoogenboom R. Schlaad H. (2011). Bioinspired poly(2-oxazoline)s. Polymers 3, 467–488. 10.3390/polym3010467

[B42] Hosseini S. Shahrousvand M. Mohammadi-Rovshandeh J. Jahanbakhshi M. Javadi A. Soleimani M. (2024). Fabrication of pH-Responsive amphiphilic poly(vinyl alcohol–methyl methacrylate) copolymer nanoparticles for application in cancer drug delivery systems. Iran. J. Sci. 48, 99–111. 10.1007/s40995-023-01573-w

[B43] Hu Z. Chen L. Betts D. E. Pandya A. Hillmyer M. A. DeSimone J. M. (2008). Optically transparent, amphiphilic networks based on blends of perfluoropolyethers and poly(ethylene glycol). J. Am. Chem. Soc. 130, 14244–14252. 10.1021/ja803991n 18834196

[B44] Kacanski M. Stelzer F. Walsh M. Kenny S. O’Connor K. Neureiter M. (2023). Pilot-scale production of mcl-PHA by Pseudomonas citronellolis using acetic acid as the sole carbon source. New Biotechnol. 78 (2023), 68–75. 10.1016/j.nbt.2023.10.003 37827242

[B45] Kalaycı Ö. A. Cömert F. B. Hazer B. Atalay T. Cavicchi K. Cakmak M. (2010). Synthesis, characterization, and antibacterial activity of metal nanoparticles embedded into amphiphilic comb-type graft copolymers. Polym. Bull. 65, 215–226. 10.1007/s00289-009-0196-y

[B46] Kalayci O. A. Duygulu O. Hazer B. (2013). Optical characterization of CdS nanoparticles embedded into the comb-type amphiphilic graft Copolymer. J. Nanoparticle Res. 15 (1355), 1355. 10.1007/s11051-012-1355-x

[B47] Karahaliloğlu Z. Ilhan E. Kılıcay E. Hazer B. Denkbas E. B. (2020). Potent bioactive bone cements impregnated with polystyrene-g-soybean oil-AgNPs foradvanced bonetissue applications. Mater. Technol. 35, 179–194. 10.1080/10667857.2019.1661157

[B48] Kılıcay E. Demirbilek M. Turk M. Guven E. Hazer B. Denkbas E. B. (2011). Preparation and characterization of poly(3-hydroxybutyrate-co-3-hydroxyhexanoate) (phbhhx) based nanoparticles for targeted cancer therapy. Eur. J. Pharm. Sci. 44 (3), 310–320. 10.1016/j.ejps.2011.08.013 21884788

[B49] Kilicay E. Erdal E. Elci P. Hazer B. Denkbas E. B. (2024). Tumour-specific hybrid nanoparticles in therapy of breast cancer. J. Microencapsul. Micro Nano Carriers 41, 45–65. 10.1080/02652048.2023.2292226 38095892

[B50] Kocer H. Borcakli M. Demirel S. Hazer B. (2003). Production of bacterial polyesters from some various new substrates by Alcaligenes eutrophus and Pseudomonas oleovorans. Turk J. Chem. 27 (3), 365–374.

[B51] Li J. Ge Z. Toh K. Liu X. Dirisala A. Ke W. (2021). Enzymatically transformable polymersome-based nanotherapeutics to eliminate minimal relapsable cancer. Adv. Mater. 33, 2105254. 10.1002/adma.202105254 34622509

[B52] Mai Y. Eisenberg A. (2012). Self-assembly of block copolymers. Chem. Rev. 41, 5969–5985. 10.1039/c2cs35115c 22776960

[B53] Mehrpouya M. Vahabi H. Barletta M. Laheurte P. Langlois V. (2021). Additive manufacturing of polyhydroxyalkanoates (PHAs) biopolymers: materials, printing techniques, and applications. Mater. Sci. Eng. C 127, 112216. 10.1016/j.msec.2021.112216 34225868

[B54] Minoda M. Sawamoto M. Higashimura T. (1990). Amphiphilic block copolymers of vinyl ethers by living cationic polymerization. 3. Anionic macromolecular amphiphiles with pendant carboxylate anions. Macromolecules 23, 1897–1901. 10.1021/ma00209a001

[B55] Miyamoto M. Naka K. Tokumizu M. Saegusa T. (1989). End capping of growing species of poly(2-oxazoline) with carboxylic acid: a novel and convenient route to prepare vinyl- and carboxy-terminated macromonomers. Macromolecules 22, 1604–1607. 10.1021/ma00194a016

[B56] Neugebauer D. Rydz J. Goebel I. Dacko P. Kowalczuk M. (2007). Synthesis of graft copolymers containing biodegradable poly(3-hydroxybutyrate) chains. Macromolecules 40, 1767–1773. 10.1021/ma062251j

[B57] Obeid R. Tanaka F. Winnik F. M. (2009). Heat-induced phase transition and crystallization of hydrophobically end-capped poly(2-isopropyl-2-oxazoline)s in water. Macromolecules 42, 5818–5828. 10.1021/ma900838v

[B58] Park J. S. Akiyama Y. Winnik F. M. Kataoka K. (2004). Versatile synthesis of end-functionalized thermosensitive poly(2-isopropyl-2-oxazolines). Macromolecules 37, 6786–6792. 10.1021/ma049677n

[B59] Park J.-S. Kataoka K. (2007). Comprehensive and accurate control of thermosensitivity of poly(2-alkyl-2-oxazoline)s via well-defined gradient or random copolymerization. Macromolecules 40, 3599–3609. 10.1021/ma0701181

[B60] Tuzen M. Sahiner S. Hazer B. (2016). Solid phase extraction of lead, cadmium and zinc on biodegradable polyhydroxybutyrate diethanol amine (PHB-DEA) polymer and their determination in water and food samples. Food Chem. 210, 115–120. 10.1016/j.foodchem.2016.04.079 27211628

[B61] Ullah R. Tuzen M. Hazer B. Saleh T. A. (2024). Synthesis of poly (3-hydroxy butyrate)-g-poly (ricinoleic acid)-Ag nanocomposite for adsorption of methyl blue with multivariate optimization. J. Mol. Liq., 124369. 10.1016/j.molliq.2024.124369

[B62] Unsal Y. E. Soylak M. Tuzen M. Hazer B. (2015). Determination of lead, copper, and iron in cosmetics, water, soil, and food using polyhydroxybutyrate-b-polydimethyl siloxane preconcentration and flame atomic absorption spectrometry. Anal. Lett. 48, 1163–1179. 10.1080/00032719.2014.971365

[B63] Vergaelen M. Monnery B. D. Jerca V. V. Hoogenboom R. (2023). Detailed understanding of solvent effects for the cationic ring-opening polymerization of 2**-**Ethyl-2-oxazoline. Macromolecules 56 (4), 1534–1546. 10.1021/acs.macromol.2c01930

[B64] Wadhwa S. K. Tuzen M. Kazi T. G. Soylak M. Hazer B. (2014). Polyhydroxybutyrate-b- polyethyleneglycol block copolymer for the solid phase extraction of Lead and copper in water, baby foods, tea and coffee samples. Food Chem. 152, 75–80. 10.1016/j.foodchem.2013.11.133 24444908

[B65] Wang M. Li Y. Zheng L. Hu T. Yan M. Wu C. (2024). Amphiphilic–zwitterionic block polymers. Polym. Chem. 15, 622–630. 10.1039/d3py01179h

[B66] Wen P. Ke W. Dirisala A. Toh K. Tanaka M. Li J. (2023). Stealth and pseudo-stealth nanocarriers. Adv. Drug Deliv. Rev. 198, 114895. 10.1016/j.addr.2023.114895 37211278

[B67] Xu R. Winnik M. A. Hallet F. R. Riess G. Croucher M. D. (1991). Light-scattering study of the association behavior of styrene–ethylene oxide block copolymers in aqueous solution. Macromolecules 24, 87–93. 10.1021/ma00001a014

[B68] Yalcin B. Cakmak M. Arkın A. H. Hazer B. Erman B. (2006). Control of optical anisotropy at large deformations in PMMA/chlorinated-PHB (PHB-Cl) blends: mechano-optical behavior. Polymer 47, 8183–8193. 10.1016/j.polymer.2006.09.051

[B69] Zhou M. Qian Y. Xie J. Zhang W. Jiang W. Xiao X. (2020). Poly(2-Oxazoline)-Based functional peptide mimics: eradicating MRSA infections and persisters while alleviating antimicrobial resistance. Angew. Chem. Int. Ed. 59, 6412–6419. 10.1002/anie.202000505 32083767

